# Cohort, gender and spatial patterns of delayed school enrolment in third-grade children from 2009 to 2025

**DOI:** 10.1038/s41598-026-61806-x

**Published:** 2026-07-29

**Authors:** Maxi Woelke, Darlene Heinen-Stach, Paula Teich, Reinhold Kliegl, Michael A. Rapp, Mareike Voigt, Mira Tschorn, Fabian Arntz

**Affiliations:** 1 Social and Preventive Medicine, Department of Sports and Health Sciences, University of Potsdam, Potsdam, Germany; 2 Division of Training and Movement Science, Department of Sports and Health Sciences, University of Potsdam, Potsdam, Germany; 3 Division of Health Psychology, Department of Education and Psychology, Freie Universität, Berlin, Germany; 4 partner site Berlin, German Center for Mental Health (DZPG), Potsdam, Germany

**Keywords:** School enrolment, Developmental delay, Rurality, Socio economic, Gender, Cohort trends, Diseases, Health care, Medical research

## Abstract

Delayed school enrolment can be an important marker for developmental delays and be associated with adverse health events. Analysing enrolment delays on an epidemiological level provides valuable insights in social, rural, socioeconomic or temporal variations. Using data from the EMOTIKON study, the influence of gender, rural and temporal variation was assessed on the frequency of children older than the key age (OTK) in a sample of 292,181 third-grade children in the Federal State of Brandenburg in Germany from 2009 to 2025. Temporal variation was analysed considering interference from significant events, specifically a change in regulation incorporating parental preferences and the onset of the SARS-CoV-2 pandemic. A generalised linear mixed model (GLMM) approach with a regression discontinuity design (RDD) revealed a substantial increase in the frequency of OTK children initiated by the change in regulation which persisted through the onset of the pandemic. The analysis further identified high regional socioeconomic deprivation, living far from metropolitan areas, living in midsize centres and being a boy as correlates of delayed school enrolment. These patterns suggests delays in school enrolment as a proxy for developmental delays to be heavily influenced by regulatory and social contexts, and at the same time highlights the importance of regional and gendered factors for school readiness assessments.

**Supplementary Information:**

The online version contains supplementary material available at 10.1038/s41598-026-61806-x.

## Introduction

Psychomotor development denotes changes in a child’s motor, cognitive, emotional and social capacities^1^, during which early childhood is considered a critical period^1,2^, and adverse inferences increase the risk of adverse health outcomes in adulthood^3,4^. A readily accessible assessment of developmental differences during early childhood is delays in school enrolment, as developmental delays are grounds to delay school enrolment^5–7^. School attendance is compulsory in most Western countries, so evaluating delayed school entry provides a valuable opportunity for population-based developmental assessments.

Delays in school enrolment are associated with disabilities or learning difficulties^8,9^ which are possibly due to early health inequalities. Several studies have shown children with delayed school entry to be more likely to have health problems such as low birth weight, hearing problems, behavioural problems and asthma^10^. They are more likely to be born prematurely^9^ and tend to score lower on academic achievement tests^9,11^. In addition, children with delays in school enrolment show lower levels of physical fitness compared to their peers who are enrolled in school on time - even compared to younger children in some fitness components^12–14^.

While the correlations between delayed school enrolment, developmental delays and early health inequalities are well established, all of them also correlate with socioeconomic and rural influences, as, for example, children from socioeconomic disadvantaged households or areas are more likely to show delays in school enrolment^15,16^. Concerning rurality, several studies have shown children from socioeconomically disadvantaged rural areas to be more likely to experience developmental delays, health problems and behavioural problems^17–19^. For example, limited access to health care and educational support services in rural areas may lead to delayed diagnoses and less early intervention, which can negatively affect children’s long-term development^20,21^. Such regional inequality effects are visible not only in health outcomes but also in school performance, as access to qualified teachers and educational support services tends to be more limited in rural areas^16,22,23^.

Aside from the association with health outcomes and moderation by socioeconomic and rural inferences, delays in school entry may also be on account of parental requests rooted in their developmental or pedagogical reasoning^24^. For example, they may intend to give their children more time to adjust to the demands of school. In Germany, the Federal State of Brandenburg, decision to delay school enrolment used to be in the responsibility of the principal and based on results from a school entrance examination (Schuleingangsuntersuchung)^6,7^. However, following political pressure from parental interest groups advocating for delays in school entry^25^, decisions on school enrolment transitioned from a decision by the school’s principal to a process additionally incorporating parental preferences in 2013^26^. Over the past two decades, there has been a general trend towards an increase in diagnosed developmental delays in German children (up to 26.5% in 5–7-year-olds according to secondary statutory health insurance data)^27^. Another important event with evidence for long term ongoing adverse effects on children’s health is the onset of the SARS-CoV-2 (covid) pandemic^28,29^. For example, negative effects on child development correlate with changes to the social environment through protective measures^30^ and with adverse health effects due to long covid^28^.

Finally, when analysing social changes across time in concert with spatial and socioeconomic conditions, gender is another impiortant factor to consider, particularly as boys are more likely to be delayed in their school enrolment compared to girls^9,10,12,13^. This difference mirrors findings from studies examining gender differences in health-related measures such as developmental disorders, mental health problems, chronic diseases and childhood disability^31–34^.

In summary, given the observed trends of an increase in developmental delays in children in Germany^27^, we hypothesized an increase in delays in school enrolment over time, accelerated through changes in regulation policy and the onset of the covid pandemic. We also expected higher levels of delays of school enrolment in rural socioeconomically disadvantaged areas in boys compared to girls.

## Results

### Cohort

**Fig. 1:**
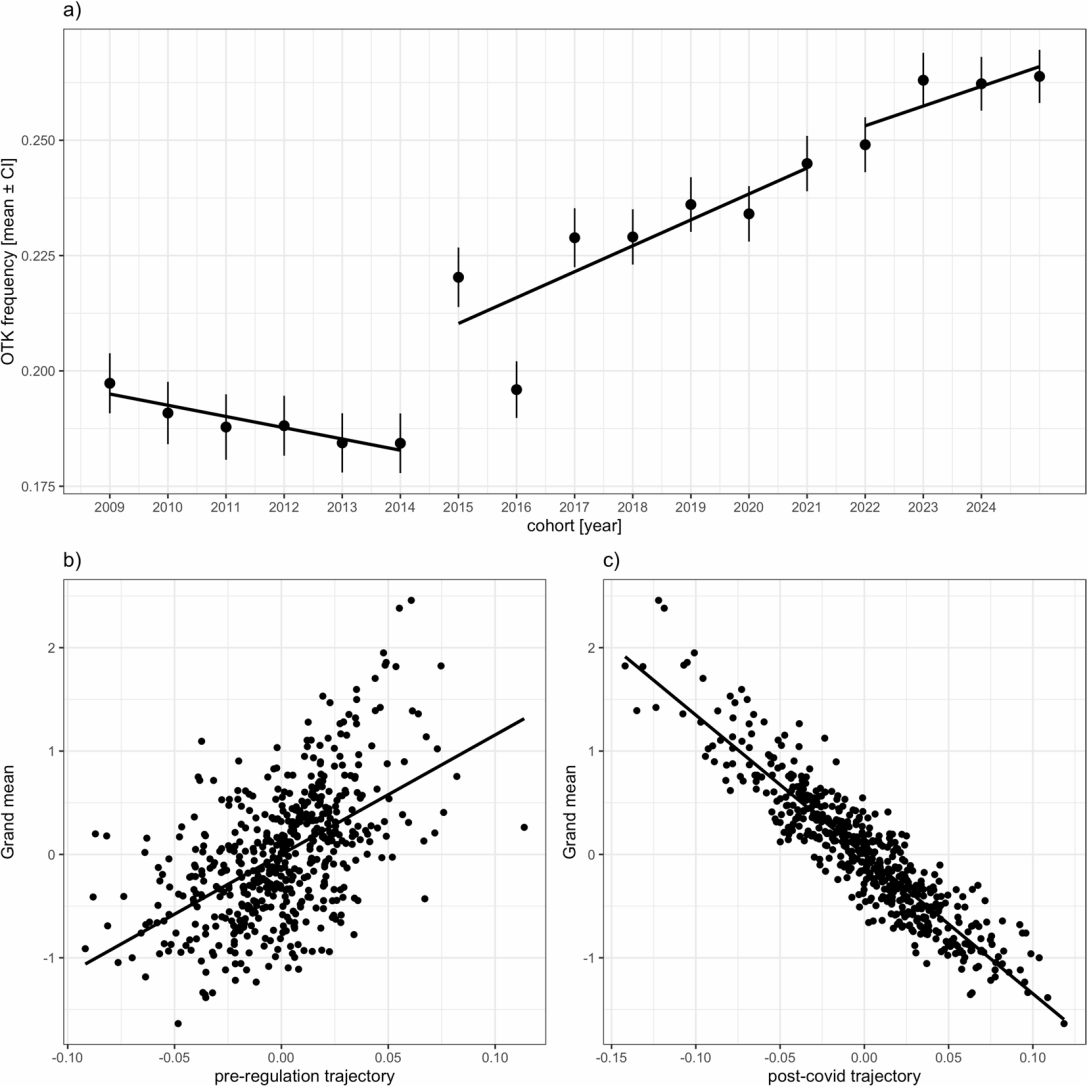
Effects of cohort trends and interactions on OTK frequencies **a**) linear cohort trends in OTK frequencies across all observed cohorts including splits for changes in regulation measurable between 2013 and 2014, and the onset on the covid pandemic measurable between 2021–2022; **b**) School level random effect correlations between grand mean and pre-regulation trajectory; **c**) School level random effect correlations between the grand mean and post-covid trajectory.

The two generalised linear mixed models revealed higher levels of OTK post regulation, compared to pre-regulation (2025-model: z = 8.50; German index of socioeconomic deprivation (GISD)-model: z = 7.68), but did not reveal a second significant increase in OTK frequencies after the onset of the covid pandemic in the first model (2025-model: z = 1.84). The regression discontinuity design (RDD) further revealed a slight decrease of OTK frequencies during the pre-regulation period, this effect however, only reached significance in model 1 (2025-model: z = -2.24; GISD-model: z = -1.95). During the post-regulation period, both models revealed clear continuous increases in OTK frequencies (2025-model : z = 7.96; GISD-model: z = 7.70). These increases further continued during the post-pandemic onset period (2025-model; z = 2.36) (see Table 1; Fig. 1).

**Table 1:** Fixed effects parameters.

	Model 1				Model 2			
	β	SE	z	*p*	β	SE	z	*p*
Grand Mean	-1.839	0.073	-25.32	< 0.001	-1.701	0.046	-36.68	< 1e-99
Covid (pre – post)	0.040	0.022	1.84	0.066				
Regulation (pre – post)	0.218	0.026	8.50	< 0.001	0.210	0.027	7.68	< 1e-13
Pre-Regulation: cohort	-0.013	0.006	-2.24	0.025	-0.012	0.006	-1.95	0.0515
Post-Regulation: cohort (pre-Covid)	0.033	0.004	7.96	< 0.001	0.063	0.008	7.70	< 1e-13
Post-Covid: cohort	0.018	0.008	2.36	0.018				
Rurality (rural area – midsize centre)	0.203	0.039	5.17	< 0.001	0.139	0.040	3.52	0.0004
Rurality (rural area – urban centre)	0.055	0.055	1.00	0.319	-0.037	0.061	-0.60	0.5501
Distance to Berlin (close – far)	0.383	0.039	9.87	< 0.001	0.310	0.048	6.49	< 1e-10
Gender (Girls – Boys)	0.486	0.020	23.98	< 0.001	0.505	0.028	17.84	< 1e-70
GISD					1.472	0.349	4.22	< 1e-04
Gender (Girls – Boys) * Rurality (rural area – midsize centre)	-0.075	0.021	-3.52	< 0.001	-0.072	0.029	-2.44	0.0148
Gender (Girls – Boys) * Rurality (rural area – urban centre)	-0.066	0.030	-2.21	0.027	-0.056	0.042	-1.35	0.1763
Gender (Girls – Boys) * Distance to Berlin (close – far)	-0.062	0.021	-2.98	0.003	-0.073	0.029	-2.53	0.0115
GISD * Regulation (pre – post)					0.099	0.311	0.32	0.7501
GISD * Pre-Regulation: cohort					-0.008	0.069	-0.12	0.9019
GISD * Post-Regulation: cohort					0.191	0.084	2.28	0.0226
GISD * Rurality (rural area – midsize centre)					-0.477	0.392	-1.22	0.2233
GISD * Rurality (rural area – urban centre)					-2.232	0.465	-4.80	< 1e-05

SE = standard error; GISD = German Index of Socioeconomic Deprivation; Model 1 = Utilising data form 2009–2025; Model 2 = utilising data from 2009–2019.

In the random effect structure of schools, the model revealed a positive correlation between the pre-regulation trajectory and the grand mean (2025-model: r = + 0.43; GISD-model: r = + 0.34) and a negative correlation between the post-covid trajectory (2025-model: *r* = -0.87), meaning schools with higher OTK frequencies showed smaller declines during the pre-regulation period and smaller increases during the post-pandemic period (see Table 2; Fig. 1). Threshold testing revealed no difference in post-covid trajectory accounting for 30, 60, or 90 days of delays (model results are provided in the online repository^35^(https://osf.io/b8v4y/files/msypz).

**Table 2:** Random effect parameters.

		Random factor						
			VC	SD	CP			
Model1								
	School							
		Grand Mean	0.583	0.763				
		Pre-Regulation: cohort	0.003	0.051	+ 0.43			
		Post-Regulation: cohort	0.002	0.044	-0.07	-0.13		
		Gender (Girls – Boys)	0.007	0.081	-0.45	-0.03	-0.11	
		Post-covid: cohort	0.005	0.069	-0.87	-0.37	-0.07	+ 0.25
	Residual		0.990	0.995				
Model2								
	School							
		Grand Mean	0.166	0.408				
		Pre-Regulation: cohort	0.002	0.052	+ 0.43			
		Post-Regulation: cohort	0.005	0.073	-0.37	-0.32		
		Gender (Girls – Boys)	0.012	0.110	-0.47	-0.14	-0.15	
	Residual		0.986	0.993				

VC = variance component (i.e., square root of variance), SD = standard deviation, CP = correlation parameter.

### Gender

**Fig. 2:**
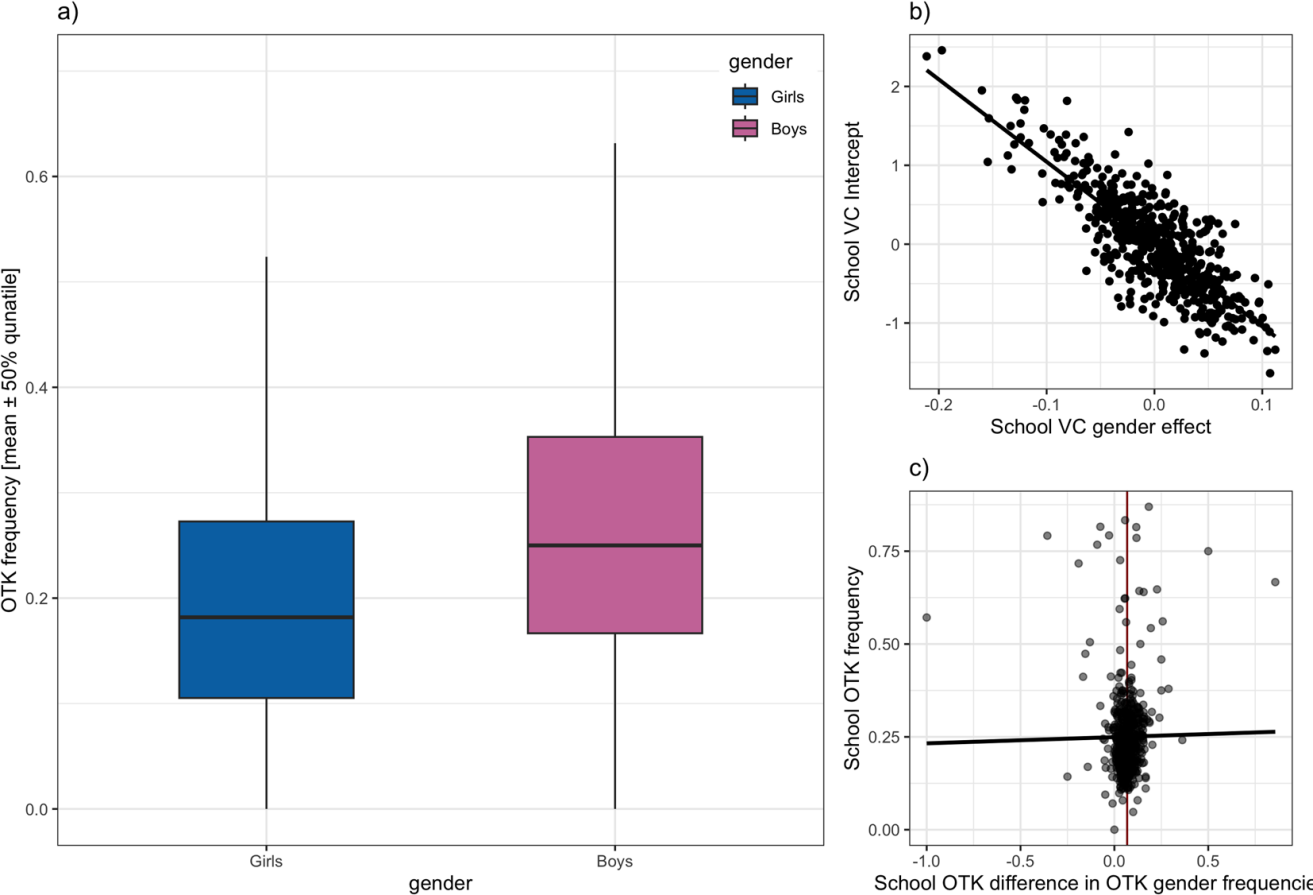
Gender differences in OTK frequencies: **a**) boxplot of differences between boys and girls in OTK frequencies; **b**) regression line plot of School level random effect correlations between the gender effect and the general OTK prevalence; **c**) School general OTK frequency to difference in gender OTK frequencies (i.e., boys OTK frequencies - girls OTK frequencies), the red horizontal line indicates the average difference in OTK frequencies.

The significant general gender effect (2025-model: z = 23.98; GISD-model: z = 17.84) revealed boys to be more likely OTK compared to girls (see Table 1; Fig. 2). In the random effect structure of school, a significant correlation between the intercept and the gender effect (2025-model: r = --0.45; GISD-model: *r* = -0.47) indicated schools with more OTK children to exhibit a smaller difference between girl and boy OTK ratios (see Table 2; Fig. 2).

### Rural regions

**Fig. 3:**
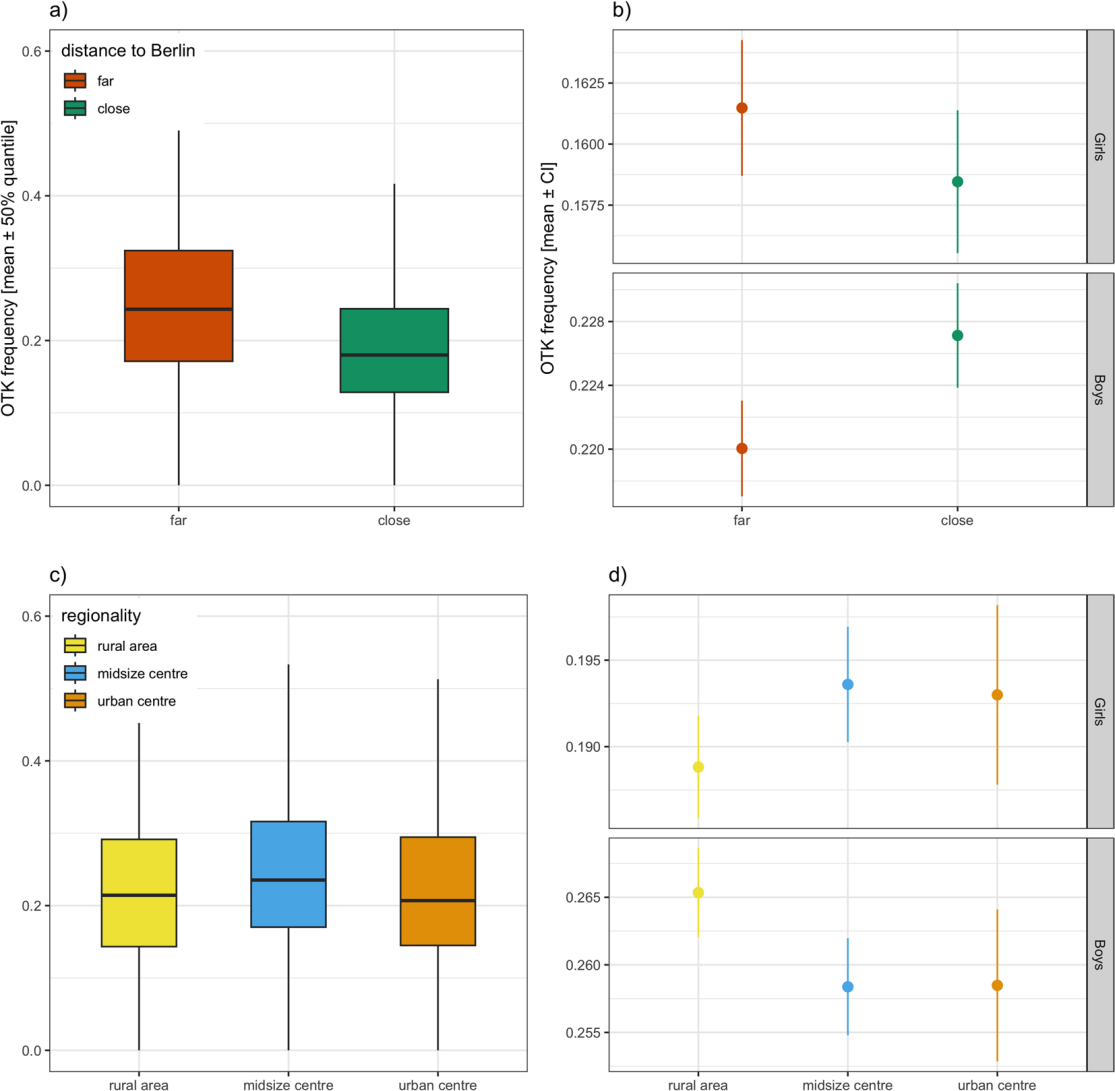
Effects of rurality of OTK frequencies. **a**) boxplot of differences between OTK frequencies close to Berlin and in the further metropolitan area in OTK frequencies; **b**) boxplot of differences between OTK frequencies urban centres, midsize centres and countryside; **c**) gendered differences between OTK frequencies close to Berlin and in the further metropolitan area in OTK frequencies (model predictions); **d**) gendered differences between OTK frequencies urban centres, midsize centres and countryside (model predictions).

In rural areas, children living further away from Berlin were more likely to be identified as OTK (2025-model: z = 9.87; GISD-model: z = 6.49). We also found children living in midsize centres more likely to be identified as OTK compared to those from rural areas (2025-model: z = 5.17; GISD-model: z = 3.52), there was no evidence for differences in OTK frequencies between rural areas and urban centres (2025-model: z = 1.00; GISD-model: z = -0.60). Additionally, we found gender differences in the effects of rurality on OTK frequencies, with girls being more likely to be identified as OTK in areas further from Berlin (compared to girls living close to Berlin), while boys were more likely to be identified in areas close to Berlin (compared to Boys living further from Berlin) (2025-model: z = -2.98; GISD-model: z = -2.53). Considering the rural to urban gradient we found girls to be least likely to be identified in rural areas, while rural boys were more likely to be identified, compared to their respective midsize centre gender counterparts (2025-model: z = -3.52; GISD-model: z = -2.44). Comparing rural areas to urban centres showed associations of an identical direction, however, no evidence for gendered differences was found in the model where regional socioeconomic deprivation variance was accounted for (2025-model: z = -2.21; GISD-model: z = -1.35) (see Table 1; Fig. 3).

### Regional socioeconomic deprivation

**Fig. 4:**
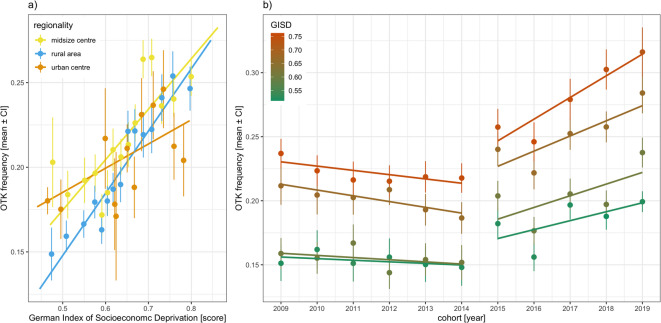
Associations of regional socioeconomic deprivation with OTK frequencies. **a**) rural differences in the association of regional socioeconomic deprivation with OTK frequencies; **b**) regional socioeconomic deprivation differences associated with the pre and post regulation cohort trends.

Analysing associations between regional socioeconomic deprivation and delays in school enrolment revealed a higher likelihood in delayed school enrolment for children from more deprived areas (GISD-model 2: z = 4.22). Further the analysis revealed a difference in slope depending on the GISD after the change in regulation (GISD-model: z = 2.28) with a steeper increase in OTK children across cohorts in areas with higher levels of socioeconomic deprivation. Finally, we found evidence for a difference in the association of the GISD depending on rural classification, with no differences between rural areas and midsize centres (GISD-model: z = -1.22) but comparing rural areas with urban centres showed a steeper increase in rural areas (GISD-model: z = -4.80) (see Table 1; Fig. 4).

Utilising the post hoc analysis implementing GISD-sub scores revealed overall associations for income (z = 5.12) and work (z = 3.94) but not education deprivation (z = 0.09). Further, the post hoc analysis revealed evidence only work deprivation to be associated with the post-regulation trajectory (z = 2.31). While evidence for differences between rural areas and urban centres in their association to OTK frequencies with regional deprivation was present for all subfactors (education: z = -2.22, work: z = -3.48, income: z = -3.74), the post hoc analyses further revealed differences in the association of regional work deprivation with OTK frequencies between rural areas and midsize centres (z = -2.60), as well (see Supplement 2 for model documentation and visualisations).

## Discussion

In the Federal State of Brandenburg, ~ 22.6% of all children between 2009 and 2025 were delayed in their school enrolment. Brandenburg has a higher rate of early school dropouts, higher levels of poverty and unemployment and overall, a lower level of education compared to the rest of Germany^36^. Triggered by the change in regulation in 2013 (i.e., including parental preferences in the school enrolment procedure), delayed school enrolment increased from 2014 to 2025 by ~ 33.7%. As expected, boys were ~ 35.4% more likely to be enrolled with a delay than girls. Regarding regional effects, OTK rates were ~ 33.8% higher in areas far from Berlin, compared to areas close to Berlin, and ~ 12.2% higher in midsize centres compared to rural areas and urban centres. Gender differences in OTK were ~ 10.1% larger in the Berlin metropolitan area and ~ 7.63% larger rural areas compared to midsize and urban centres. Considering regional socioeconomic deprivation, we found higher frequencies in school enrolment in socioeconomically disadvantaged regions. Further we found a steeper increase in OTK frequencies following the regulation change in socioeconomically disadvantaged areas, which seems to be driven primarily by lower levels of regional work-related resources. Finally, our analysis revealed reduced influences from socioeconomic deprivation in urban centres compared to rural spaces.

We found evidence for a continuous increase in OTK frequencies following the incorporation of parental preferences in the school enrolment process. Interestingly, there is evidence for an overall increase in documented developmental disorders by health insurance companies in Germany from 2007 to 2017^27^. Considering these trends, a similar increase in school enrolment delays could be expected. However, our analysis only found evidence for a comparable increase after the incorporation of parental preferences. This raises the question how parental preferences might relate to developmental disorders and how they might affect decisions on school enrolment. Pre-regulation, developmental delays were assessed using tools predominantly focussing on cognitive domains^7^. Accordingly, it could be speculated that parental preferences might be informed by non-cognitive symptoms of developmental disorders following ICD-10 classifications^27^. This could have expanded scope of developmental domains considered during the process of school enrolment which then mirrors the concurrent increase in diagnosed of developmental disorders^27^.^277^ Another possible influence are parental preferences unrelated to developmental delays, such as family and community resources supporting the child’s adaptation to the school environment^37^. They could be linked to the steeper post-regulation increase in OTK frequencies. Especially in areas with high levels of work deprivation, families are more likely to experience job insecurities^38^. Consequently, costs associated with school enrolment^39^ may be of greater concern and lead such families to opt for a delay in school entry.

After the onset of the covid pandemic OTK frequencies continuedto increase, following the post-regulation trajectory. Post hoc analyses were implemented to assess differences in slope for pre- and post-pandemic trajectories, which yielded no evidence for a difference in slope (see online repository^35^. Unfortunately, the analysis does not allow for a differentiation if the post-pandemic increase as a continuation of the post-regulation increase or if covid-pandemic associated factors further increase the OTK frequencies. However, as a recent study reported prevalences of long covid symptoms such as fatigue, post exertional malaise, or cognitive dysfunction between 10 to 20% of all children by August 2023^40^, more research is needed to elucidate the effects of the ongoing covid pandemic on child development and timing of school enrolment. Considering the random effect correlation, schools with lower OTK frequencies showed steeper increases during the covid-pandemic. Assuming a ‘soft cap’ to delays in school enrolment, there was more ‘room’ for an increase in OTK frequencies in areas where the option of delayed enrolment had not been used as much before.

Assessing rural and regional socioeconomic influences revealed higher frequencies of OTK in areas further away from Berlin, in midsize centres and in socioeconomically disadvantaged areas (especially low in work and income resources). These findings mostly coincide with studies showing children from families with lower socioeconomic status exhibiting higher rates of developmental delays^16,41,42^. Lower levels of individual as well as regional socioeconomic resources may limit access to health and educational resources^43,44^, leading to children’s delayed development. In this context, we were did not expect to find the highest levels of delays in school enrolment in midsize centres, with no differences between urban centres and rural areas. Higher cost of living in midsize centres (compared to urban areas)^45^ without the benefit of urban educational and health care infrastructure^43,44^, may create an environment uniquely challenging to child development. However, this is a speculation in need of research dissociating these correlations.

Finally, the analysis also showed a well-established higher OTK frequency for boys than girls^6,46,47^. This finding is also consistent with findings from gendered distribution in prevalences of developmental disabilities^31–33^ which might likely be associated with delays in school enrolment or at least be subject to similar influences. For example, gendered referral and detection bias^48^ could facilitate the identification of developmental delays in boys^31,49,50^. For example, for girls to be diagnosed with ADHD, they must display additional behaviours and emotional problems compared to boys^51^. Similarly, the assessment of developmental delays in girls might only identify girls if the display a wider range of developmental delays compared to boys.

In summary, delays in school enrolment arise from developmental, pedagogical, and social gradients and vary depending in socioeconomical, rural and cultural context. They can also be subject to different identification biases. Our study specifically highlights the need to further elucidate the relationship between developmental delays and delays in school enrolment and its moderators. More research is needed to identify challenges to child development prevalent in midsize centres and to elucidate the gendered gap in delayed school enrolment.

## Methods

### Methodological approach

Data used in this analysis was obtained from the EMOTIKON project (uni-potsdam.de/en/emotikon/), whose main objective is the annual assessment of physical fitness in third grade children in the Federal State of Brandenburg, Germany and is mandatory for public primary schools. The project was commissioned and approved by the Ministry of Education, Youth and Sports of the Federal State Brandenburg^52^. Data was anonymised by the Ministry of Education, Youth and Sport of the Federal State of Brandenburg; data acquisition and analyses occurred in accordance with the current Declaration of Helsinki^53^ and the Brandenburg School Act^54^.

The analysis utilises data from 2009 to 2025. Parts of the dataset have been published previously, including cohorts from 2011 to 2019^12,55^ and cohorts from 2016 to 2023^13,56^. Analysis scripts and data are further documented in the Supplement and in the Open Science Framework (OSF) repository^35^.

### Population

This repeated cross-sectional analysis assesses the data from 550 schools across 17 cohorts (i.e. 2009–2025). Delays in school enrolments was defined as a binary (dummy) operator using the enrolment date of September 30th to identify children as key age (i.e., age ≤ 6 years and 11 months at the date of enrolment) or older-than-key age (OTK) (i.e., age > 6 years and 11 months at the date of enrolment)^12^. Accordingly, 292,181 children were included in the analysis; 66,188 of them were OTK. The number of schools, communes and children per cohort are presented in Table 3. The implemented assessment of the children’s gender was done using the information provided by the schools which utilise the documented gender assigned at birth.

**Table 3:** Cohort description.

Cohort	School	Child	Gender	Key age
	[*N*]	[*N*]	[% Girls]	[% OTK]
2009	388	14,332	49.0	19.73
2010	358	12,977	49.2	19.09
2011	322	11,622	48.3	18.78
2012	381	13,874	48.5	18.81
2013	382	14,013	49.6	18.44
2014	362	13,802	48.4	18.43
2015	406	15,856	49.4	22.03
2016	386	16,035	49.6	19.59
2017	406	16,573	48.5	22.89
2018	452	18,848	48.8	22.90
2019	475	19,745	49.0	23.61
2020	461	19,039	49.6	23.40
2021	477	19,731	48.3	24.49
2022	473	20,267	49.4	24.90
2023	474	21,159	48.9	26.30
2024	475	21,892	48.6	26.22
2025	474	22,416	49.5	26.38
all	550	292,181	49.0	22.70

N = absolute number, OTK = older than keyage.

### Cohort trends

The date of school enrolment, defined as the first day of the first grade, was determined for each third-grade child retrospectively based on the year of assessment. In addition, two exact dates were implemented for the analysis to assess the change in regulation and the onset of the covid pandemic. The change in the regulation, which included parental preferences in the school enrolment process, was implemented on 29th of November 2013^26^. The onset of the covid pandemic was determined on the 30th of January 2020 at the declaration of the Public Health Emergency of International Concern by the WHO^29^.

### Regional variation

Regional variation focussing rural differences were assessed utilising two variables based on the *“Landesentwicklungsplan”*^57^ of Brandenburg, county. First, areas were classified into urban centres [*Oberzentrum*], midsize centres [*Mittelzentrum*], and rural areas [*Land*], Urban centres are defined as regional centres providing facilities to cover the specialised higher demand for a larger area. Midsize centres are defined as smaller regional centres providing facilities to meet the higher needs of the inhabitants of their central area and rural areas are regions where none of the above applies. Additionally, due to the Federal State of Brandenburg surrounding Berlin, we distinguished between the region close to vs far away from the metropolitan area of Berlin^58^. Regional variation focussing socioeconomic differences were operationalised with the German Index for Socioeconomic Deprivation (GISD). The GISD is a composite index, comprised of three subfactors assessing regional education, work and income deprivation and is available until 2019^38^. Distributions of schools, children and mean socioeconomic deprivation across the two rurality classifications are documented in Table 4 and maps for illustration of spatial rural and socioeconomic distributions are provided in the supplement (Figure S1) as well as the online repository^35^(https://osf.io/b8v4y/files/msypz).

**Table 4:** Regional distribution.

Region	Berlin distance	School	Child	Gender	Key age	GISD*
		[*N*]	[*N*]	[% Girls]	[% OTK]	[score]
rural areas	close	69	52,953	49.0	17.2	0.58
	far	199	78,181	49.1	24.4	0.66
midsize centres	close	71	47,846	48.7	20.3	0.60
	far	133	68,844	49.2	27.1	0.70
urban centres	close	37	19,479	48.4	19.9	0.47
	far	41	24,878	49.2	23.4	0.69

N = absolute number, OTK = older than keyage, K = keyage; GISD = German index for socioeconomic deprivation; *estimates averaged for cohorts 2009–2019.

### Generalised linear mixed model (GLMM)

Two generalised linear mixed models (GLMMs) with a Bernoulli distribution implementing a regression discontinuity design (RDD) were chosen to assess effects of cohort trends, regional differences, and gender on OTK frequencies. The first model (2025-model) specified also a two-threshold RDD implementing the change in regulation and the onset of the covid pandemic with the exact dates of school enrolment from 2009 to 2025 as running variables. As changes in regulation were applied to all children, no threshold testing was implemented for the regulation discontinuity. For the onset of the covid pandemic, several additional thresholds were tested (+ 30/60/90 days). The GISD was not included in this model. The second model implemented a single threshold RDD for the time from 2009 to 2019 and included the GISD in the analysis (GISD-model). For further verification of the robustness of the threshold chosen for the onset of the Covid pandemic, a model single threshold RDD design, omitting pre-regulations data was estimated and reported in the online material.

Sequential difference contrasts were used for binary variables such as gender (girl - boys), distance to Berlin (far – close), regulation (pre - post) and covid (pre - post). Custom contrasts followed a sequential differences structure comparing rural areas with midsize centres (-1,1,0) and rural areas with urban centres (-1,0,1). Interactions between the GISD and the RDD variables (i.e., running variables and exact dates for change in regulation) were included in the second model.

Following a parsimonious model selection strategy^59^, interactions between gender and both rurality variables were included in both models. The interaction between GISD and Region was added to the second model^62^. The model selection process is reported in the supplementary material and OSF repository^35^.

Concerning the random-effect structure, school was included as a random factor in all modes. Random effect estimation for Schools included the RDD variables derived from exact dates and running variable, as well as gender.

### Software

Data preprocessing and visualisation was done using the *tidyverse*^60^ and *easystats*^61^ packages in the R language version 4.2.2^62^ and RSudio IDE Version 2023.06.0 + 421^63^. GLMMs were estimated and post-processed in Julia Version 1.12.2 and Visual Studio Code Version 1.83.1 using the *MixedModels.jl*^64^ and *MixedModelsExtras.jl* package^65^.

## Supplementary Information

Below is the link to the electronic supplementary material.

Supplementary Material 1

## Data Availability

Supplementary material, data, as well as R and Julia scripts are available in the Open Science Framework (OSF) repository: [https://osf.io/b8v4y](https:/osf.io/b8v4y).
