# Supplementary material for: Cohort, gender and spatial patterns of delayed school enrolment in third-grade children from 2009 to 2025
Source: Sci Rep. 2026 Jul 29;16:23543. doi: 10.1038/s41598-026-61806-x (PMC13421696; doi:10.1038/s41598-026-61806-x)
Supplement: Supplementary file 1 — Supplementary Material 1 [file 41598_2026_61806_MOESM1_ESM.docx]

Supplementary material

Supplement 1


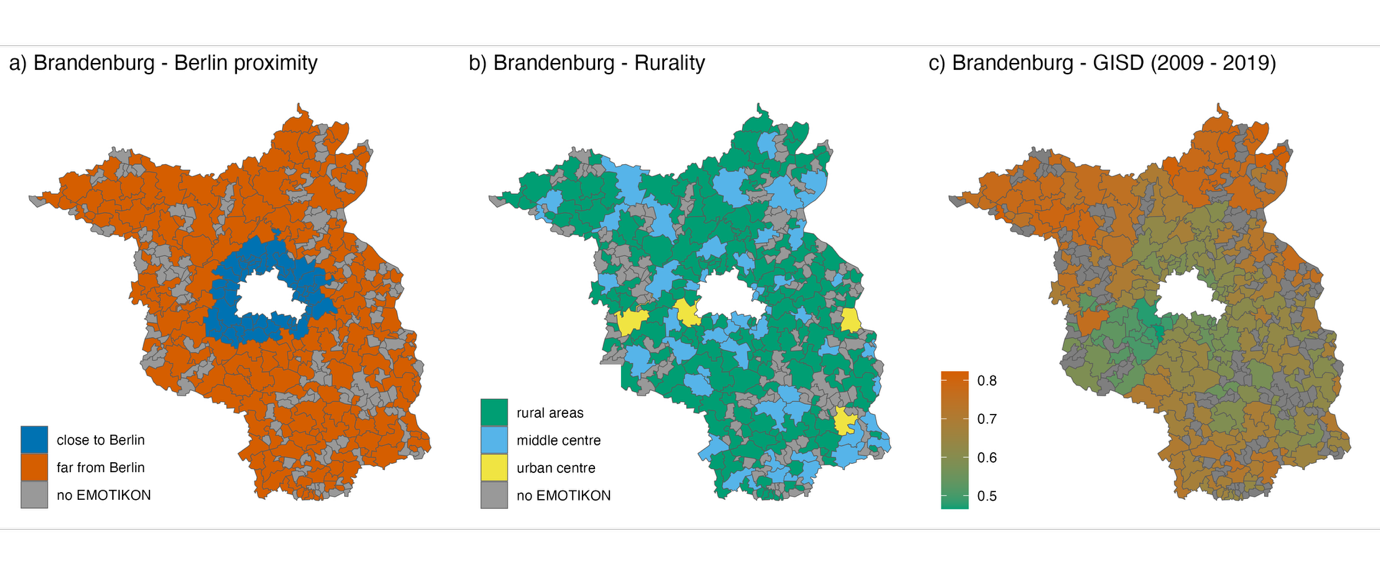
Figure S1: Illustration of spatial patterns of Brandenburg a) classification of areas close to Berlin and far from Berlin, b) classification of areas into urban areas, middle centers and urban centers, & c) classification according to German index of Socioeconomic Deprivation (mean score from 2009-201

Supplement 2

*Table S1*: Fixed effects parameters of post hoc analyses models utilising GISD sub scores

|  | Education deprivation | | | | Work deprivation | | | | Income deprivation | | | |
| --- | --- | --- | --- | --- | --- | --- | --- | --- | --- | --- | --- | --- |
|  |  |  | z | p |  |  | z | p |  |  | z | p |
| Grand mean | -1.79 | 0.04 | -41.64 | <0.001 | -1.69 | 0.05 | -37.45 | <0.001 | -1.63 | 0.05 | -34.58 | <0.001 |
| Regulation (pre – post) | 0.20 | 0.03 | 6.88 | <0.001 | 0.18 | 0.03 | 6.50 | <0.001 | 0.19 | 0.03 | 6.68 | <0.001 |
| Pre-Regulation:cohort | -0.01 | 0.01 | -1.74 | 0.082 | -0.01 | 0.01 | -0.78 | 0.4343 | 0.01 | 0.01 | 1.23 | 0.2201 |
| Post-Regulation:cohort | 0.04 | 0.01 | 5.62 | <0.001 | 0.06 | 0.01 | 7.27 | <0.001 | 0.07 | 0.01 | 7.94 | <0.001 |
| Rurality (rural area – midsize center) | 0.18 | 0.04 | 4.30 | <0.001 | 0.13 | 0.04 | 3.16 | 0.0016 | 0.16 | 0.04 | 4.01 | <0.001 |
| Rurality (rural area – urban center) | -0.06 | 0.07 | -0.84 | 0.402 | -0.01 | 0.06 | -0.24 | 0.8119 | -0.03 | 0.06 | -0.44 | 0.6570 |
| Distance to Berlin (close – far) | 0.43 | 0.04 | 10.04 | <0.001 | 0.33 | 0.05 | 7.09 | <0.001 | 0.24 | 0.05 | 5.03 | <0.001 |
| Gender (Girls – Boys) | 0.50 | 0.03 | 17.67 | <0.001 | 0.50 | 0.03 | 17.72 | <0.001 | 0.50 | 0.03 | 17.66 | <0.001 |
| Deprivation | 0.02 | 0.24 | 0.09 | 0.929 | 0.98 | 0.25 | 3.94 | <0.001 | 1.58 | 0.31 | 5.12 | <0.001 |
| Gender (Girls – Boys) * Rurality (rural area – midsize center) | -0.07 | 0.03 | -2.30 | 0.022 | -0.07 | 0.03 | -2.37 | 0.0176 | -0.07 | 0.03 | -2.34 | 0.0192 |
| Gender (Girls – Boys) * Rurality (rural area – urban center) | -0.05 | 0.04 | -1.16 | 0.247 | -0.06 | 0.04 | -1.32 | 0.1865 | -0.05 | 0.04 | -1.31 | 0.1894 |
| Gender (Girls – Boys) * Distance to Berlin (close – far) | -0.07 | 0.03 | -2.49 | 0.013 | -0.07 | 0.03 | -2.46 | 0.0140 | -0.07 | 0.03 | -2.42 | 0.0154 |
| Deprivation * Regulation (pre – post) | -0.10 | 0.24 | -0.41 | 0.680 | 0.23 | 0.23 | 0.98 | 0.3289 | -0.00 | 0.29 | -0.00 | 0.9965 |
| Deprivation * Pre-Regulation:cohort | 0.04 | 0.05 | 0.67 | 0.506 | -0.07 | 0.05 | -1.37 | 0.1718 | -0.05 | 0.07 | -0.81 | 0.4186 |
| Deprivation * Post-Regulation:cohort | 0.11 | 0.06 | 1.71 | 0.087 | 0.16 | 0.07 | 2.31 | 0.0207 | 0.11 | 0.08 | 1.42 | 0.1548 |
| Deprivation * Rurality (rural area – midsize center) | 0.18 | 0.29 | 0.64 | 0.523 | -0.68 | 0.26 | -2.60 | 0.0092 | -0.25 | 0.31 | -0.81 | 0.4157 |
| Deprivation * Rurality (rural area – urban center) | -0.73 | 0.33 | -2.22 | 0.026 | -1.69 | 0.48 | -3.48 | <0.001 | -1.37 | 0.37 | -3.74 | <0.001 |
|  |  |  |  |  |  |  |  |  |  |  |  |  |

*Table S2*: Random effects parameters of post hoc analyses models utilising GISD sub scores

|  | Random factor | | VC | SD | CP |  |  |
| --- | --- | --- | --- | --- | --- | --- | --- |
| Education deprivation | | | | | | | |
|  | School | |  |  |  |  |  |
|  |  | Grand Mean | 0.172 | 0.416 |  |  |  |
|  |  | Pre-Regulation:cohort | 0.003 | 0.051 | +0.40 |  |  |
|  |  | Post-Regulation:cohort | 0.006 | 0.075 | -0.35 | -0.35 |  |
|  |  | Gender (Girls – Boys) | 0.012 | 0.109 | -0.44 | -0.14 | -0.15 |
|  | Residual | | 0.986 | 0.993 |  |  |  |
| Work deprivation | | | | | | | |
|  | School | |  |  |  |  |  |
|  |  | Grand Mean | 0.166 | 0.408 |  |  |  |
|  |  | Pre-Regulation:cohort | 0.003 | 0.051 | +0.45 |  |  |
|  |  | Post-Regulation:cohort | 0.005 | 0.073 | -0.34 | -0.29 |  |
|  |  | Gender (Girls – Boys) | 0.012 | 0.108 | -0.43 | -0.13 | -0.16 |
|  | Residual | | 0.986 | 0.993 |  |  |  |
| Income deprivation | | | | | | | |
|  | School | |  |  |  |  |  |
|  |  | Grand Mean | 0.164 | 0.405 |  |  |  |
|  |  | Pre-Regulation:cohort | 0.003 | 0.052 | +0.44 |  |  |
|  |  | Post-Regulation:cohort | 0.006 | 0.075 | -0.36 | -0.31 |  |
|  |  | Gender (Girls – Boys) | 0.012 | 0.109 | -0.44 | -0.11 | -0.13 |
|  | Residual | | 0.986 | 0.993 |  |  |  |


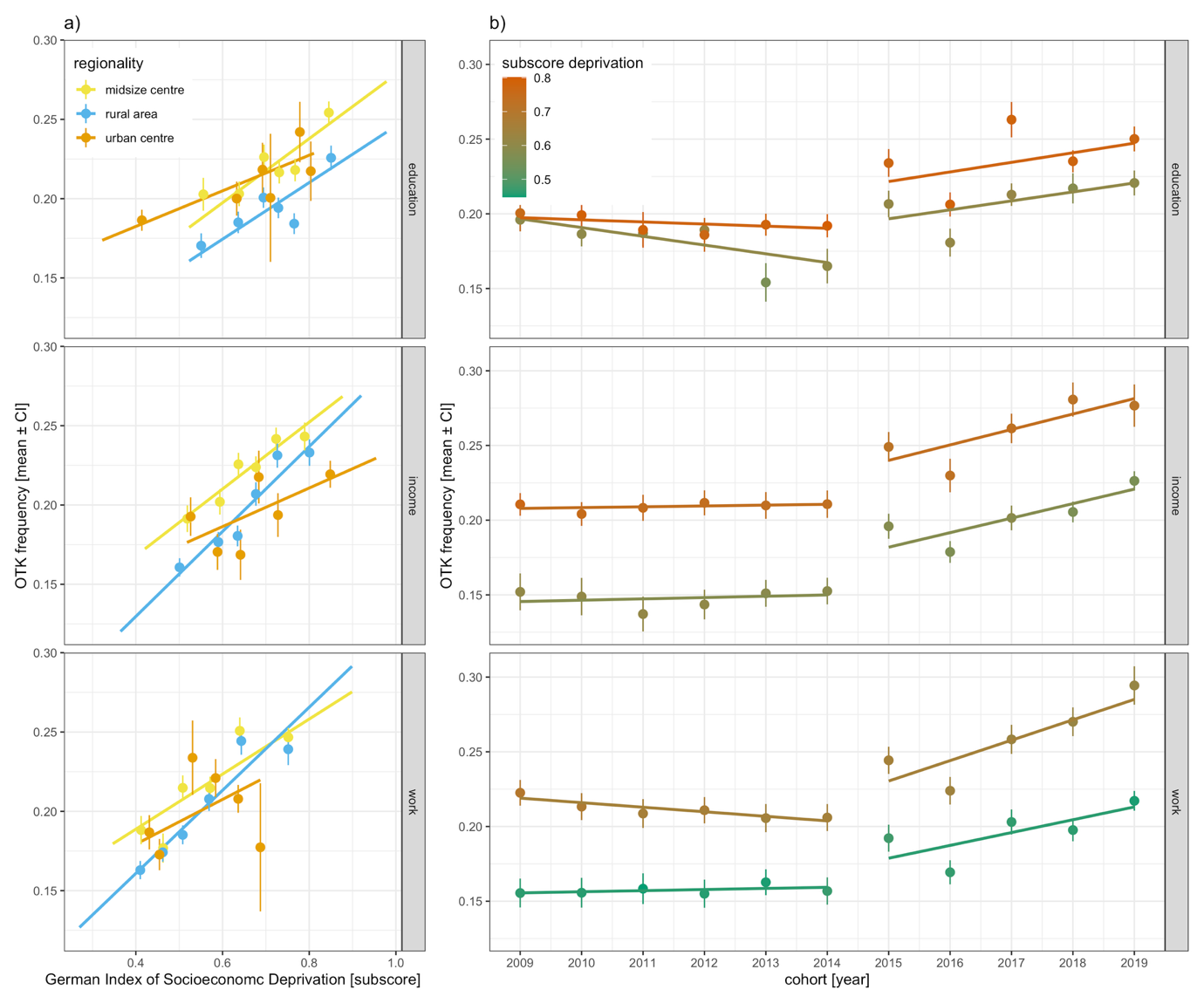


Figure S2: Associations of regional education, work and income deprivation with OTK frequencies. a) rural differences in the association of regional education, work and income deprivation with OTK frequencies; b) regional education, work and income deprivation differences associated with the pre and post regulation cohort trends, grouping for illustration of deprivation utilised subscore specific median splits
